# The association of delta neutrophil index with the prognosis of acute exacerbation of chronic obstructive pulmonary disease

**DOI:** 10.1186/s12890-020-1083-4

**Published:** 2020-02-19

**Authors:** Sunmin Park, Sang Jun Lee, Beomsu Shin, Seok Jeong Lee, Sang-Ha Kim, Woo Cheol Kwon, Jihye Kim, Myoung Kyu Lee

**Affiliations:** 1 Department of Internal Medicine, Yonsei University Wonju College of Medicine, 20, Ilsan-ro, Ilsan-dong, Wonju-si, Gangwon-do 26426 South Korea; 2 Department of Radiology, Yonsei University Wonju College of Medicine, Wonju, Gangwon South Korea; 3 Division of Pulmonary, Critical Care, Sleep and Allergy, Department of Medicine, University of Illinois at Chicago, Chicago, Illinois USA; 4 Department of Evidence based medicine, Yonsei University Wonju College of Medicine, Wonju, Gangwon South Korea

**Keywords:** Acute exacerbation, Chronic obstructive pulmonary disease, Community-acquired pneumonia, Delta neutrophil index, Mortality, Readmission

## Abstract

**Background:**

Acute exacerbations of chronic obstructive pulmonary disease (AECOPD) is associated with infective triggers including bacterial or viral in many cases, and pneumonia is a major contributor to hospitalization for AECOPD and has a close relationship with poor outcomes. Increased delta neutrophil index (DNI) can be useful in the detection of COPD patients with pneumonia.

**Methods:**

A retrospective cohort study was performed to investigate the mortality rate of the patients who were re-admitted within 6 months after discharge from the hospital due to AECOPD with or without CAP. We analyzed the difference of cumulative survival rate according to serum DNI level and readmission duration.

**Results:**

Finally, 140 AECOPD patients with community-acquired pneumonia (CAP) and 174 AECOPD patients without CAP were enrolled during 6 months, respectively. The mean age was 72.2 ± 9.4 year-old, and 240 patients (76.4%) were male. When comparing the cumulative survival rate according to readmission duration (≤ 30 vs >  30 days) and DNI level (< 3.5 vs ≥ 3.5%), AECOPD patients with readmission ≤30 days and DNI ≥ 3.5% showed the lowest cumulative survival rate compared to other groups (*P* <  0.001). Multivariate analysis revealed readmission duration ≤30 days (HR 7.879, 95% CI 4.554–13.632, *P* <  0.001); and serum DNI level (HR 1.086, 95% CI 1.043–1.131, P <  0.001) were significantly associated with the mortality of AECOPD patients during 6 months. The area under the curve for readmission (≤ 30 days) + DNI level (≥ 3.5%) was 0.753 (95% CI 0.676–0.830, *P* <  0.001) with a sensitivity of 73.7% and a specificity of 67.3%.

**Conclusion:**

AECOPD patients who were readmitted ≤30 days and DNI ≥ 3.5% showed higher mortality. DNI level can be used as a predictor of prognosis in AECOPD patients who were readmitted after discharge.

## Background

Acute exacerbations of chronic obstructive pulmonary disease (AECOPD) are major health issues in COPD patients, and are important causes of hospital admission and mortality [1–3]. AECOPD is diagnosed on clinical grounds, when specific symptoms (including dyspnea, increased sputum volume, and purulence) deteriorate beyond day-to-day variability, whereas severity is rated according to healthcare resource utilization [4].

The observational study showed that the readmission for AECOPD within 30 days is associated with a progressive increased long-term risk of death [5]. Nationwide study also demonstrated that 30-day readmissions after an AECOPD remain a major healthcare burden, associated with both patient and clinical factors (longer length of stay and discharge to a skilled nursing facility) [6]. Most exacerbations appear to be associated with infective triggers including bacterial or viral causatives [3]. Increased frequency of exacerbations is also significantly associated with forced expiratory volume in 1 s (FEV_1)_ decline [7], and can thereby increase disease severity and mortality [8].

Community-acquired pneumonia (CAP) is a frequently accompanied disease [9] and is a valuable predictive factor of poor prognosis in AECOPD patients who require hospitalization [10]. AECOPD patients with pneumonia were found to use non-invasive ventilation more frequently and remain hospitalized longer than those without pneumonia [11]. Several studies have identified that old age, disease severity, and use of inhaled corticosteroids are predisposing factors better inducing CAP in COPD patients [10, 12]. There is also another problem that readmission of AECOPD patients discharged after inpatient treatment. In fact, some studies explained that about 20% were readmitted due to AECOPD within 30 days after discharge [13, 14].

The delta neutrophil index (DNI) is the immature granulocyte fraction determined by subtracting the fraction of mature polymorphonuclear leukocytes and reflects the number of immature neutrophils as a blood biomarker [15]. DNI can easily be calculated and reported without an additional cost. Recently, systematic review and meta-analysis showed that the DNI has prognostic value in adults with sepsis and high DNI values tended to be associated with mortality in septic patients [16]. Increased DNI can be useful to evaluate the prognosis of COPD patients, especially with pneumonia. However, many studies about on AECOPD patients who were readmitted after receiving inpatient treatment and discharge have not yet been published and there is no study of the relationship between DNI and AECOPD prognosis.

Therefore, we performed a retrospective cohort study to investigate the mortality rate of AECOPD patients with or without CAP who were readmitted within 6 months after discharge from the hospital. Then, we analyzed the cumulative survival rate according to serum DNI level and readmission duration of AECOPD patients.

## Methods

### Subjects

**Fig. 1:**
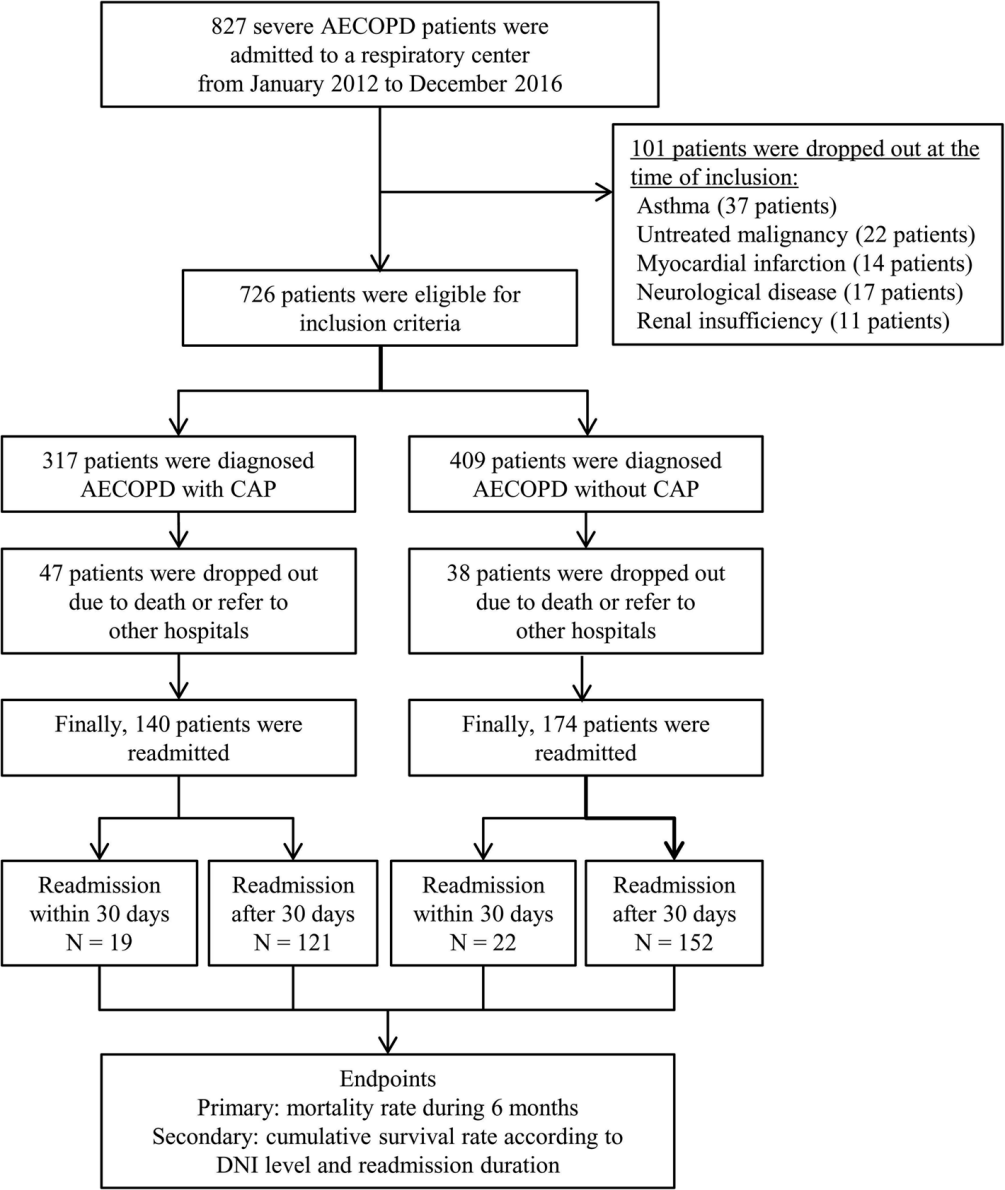
Flowchart shows identification of severe AECOPD patients who were admitted to a respiratory center. AECOPD = acute exacerbation of chronic obstructive pulmonary disease; CAP = community-acquired pneumonia; DNI = delta neutrophil index

We conducted a retrospective study of the patients who admitted with AECOPD at Yonsei University Wonju Severance Tertiary Hospital from January 2012 to December 2016 (Fig. 1). Patients were 45 years old or older and had a smoking history of ≥10 pack-years.

The diagnostic criteria for COPD were as follows according to the GOLD guideline; a post-bronchodilator FEV_1_/forced vital capacity (FVC) < 70% confirms airflow limitation that is not fully reversible [1, 3]. We reviewed demographic data and comorbidities including diabetes mellitus, cardiac, liver and renal diseases and also investigated the treatments for COPD including long-acting muscarinic antagonists, beta_2_ agonist, and inhaled corticosteroids. We evaluated lung function via using the Korean language version of the COPD assessment test (CAT) questionnaire [17], the modified Medical Research Council (mMRC) dyspnea scale, and most recent spirometry performed before admission, respectively. The following patients were excluded; valvular heart disease, myocardial infarction, cerebral infarction or hemorrhage, asthma, untreated malignancy, and renal disease on hemodialysis.

### Admission criteria

AECOPD can be diagnosed when a patient with COPD experiences a sustained (24–48 h) increase in cough, sputum production, and/or dyspnea [18]. The admission criteria of AECOPD were as follows; 1) failed response to initial medical management, 2) severe symptoms (resting dyspnea, respiratory rates ≥ 30 breaths/min, oxygen saturation ≤ 90%, confusion, or drowsiness), 3) new onset of cyanosis, peripheral edema, or 4) respiratory failure using respiratory muscles or accompanying mental changes [19].

CAP was diagnosed when the following conditions were met; 1) cough and at least one other lower respiratory tract symptom; 2) new focal chest signs on examination; 3) at least one systemic feature of sweating, fevers, aches and and/or temperature ≥ 38 °C) [20]. The causative organism was recognized when detected in sputum or bronchoalveolar lavage fluid and/or blood. Sputum specimens were recognized when > 25 leukocytes and < 10 epithelial cells per high power field [21]. We performed peripheral blood sampling within 1 hour of admission to verify white blood cell (WBC) counts, delta neutrophil index (DNI), hemoglobin, high sensitive C-reactive protein (hs-CRP) and procalcitonin. Oxygen saturation, partial pressure of oxygen (PaO2) and carbon dioxide (PaCO2) were measured on day 1.

### Treatments and endpoint

We treated enrolled patients with nebulized salbutamol, ipratropium bromide, Budesonide and intravenous prednisolone in a dosage of 30 to 40 mg daily, according to GOLD guidelines [1]. Systemic corticosteroid was given during 10–14 days, and switched to an oral prednisolone on day 4–7. Antibiotics were used in patients with CAP and adjusted according to antimicrobial susceptibilities on sputum or blood culture analysis. Antibiotic therapy was initiated in basic accordance with the ATS/IDSA guidelines [22].

We investigated the mortality rate of AECOPD patients with or without CAP who were readmitted within 6 months after discharge from the hospital. We analyzed cumulative survival rate according to serum DNI level (< 3.5 vs ≥ 3.5%) and readmission duration (≤ 30 vs >  30 days) in AECOPD patients.

### Statistical analysis

SPSS 24.0 (SPSS Inc.; Chicago, IL, USA) were used for statistical analysis. Chi-square or Fisher’s exact test were used for categorical variables and Student *t* or Mann-Whitney U test used for continuous variables. We used Cox proportional hazards regression model to estimate the survival rate during 6 months between two groups. Relative risks were expressed as hazard ratio (HR) and 95% confidence interval (CI). Cumulative survival rates were expressed using a Kaplan–Meier approach and the log-rank test. Univariate and multivariate analysis was performed to evaluate prognostic factors associated with mortality of the patients. The receiver operating characteristic (ROC) curve was utilized to assess the accuracy of different indicators for mortality of AECOPD. We compared the area under the ROC curve (AUC) according to 1) readmission duration, 2) DNI, 3) readmission duration + DNI, and 4) readmission duration + CAP. The cut-off value for DNI was set at 3.5%. Pearson’s correlation coefficients (r value) test was used for evaluating the relationship between two variables. Descriptive statistics were expressed as mean value ± standard deviation for continuous data and number (%) for categorical data. *P-*value less than 0.05 were considered to be statistically significant.

## Results

### Total subjects

During study period, 827 severe AECOPD patients were admitted to a respiratory center, and 726 patients were eligible for inclusion criteria. Eighty-five patients (47 patients with CAP and 38 patients without CAP) were dropped out because were referred to other hospitals. Finally, 140 AECOPD patients with CAP (19 patients who were readmitted within 30 days and 121 patients who were readmitted after 30 days) and 174 AECOPD patients without CAP (22 patients who were readmitted within 30 days and 152 patients who were readmitted after 30 days) were enrolled during 6 months, respectively (Fig. 1). The mean age was 72.2 ± 9.4 year-old, and 240 patients (76.4%) were male. The demographic characteristics of AECOPD patients who were readmitted between ≤30 days and > 30 days are shown in Table 1. Underlying comorbid conditions except hypertension (*P* = 0.042), the regular inhaled medications and spirometric results before an admission were not significantly different among four groups (Table 1).

**Table 1:** Characteristics of AECOPD patients with and without CAP

Characteristics	AECOPD with CAP		*P*-value	AECOPD without CAP		*P*-value
	Readmission ≤ 30 days (*n* = 19)	Readmission > 30 days (*n* = 121)		Readmission ≤ 30 days (*n* = 22)	Readmission > 30 days (*n* = 152)	
Age, y, mean (SD)	76.2 ± 7.4	72.1 ± 8.7	0.055	72.8 ± 11.1	71.8 ± 9.8	0.655
Male sex, n (%)	14 (73.7)	93 (76.9)	0.627	15 (68.2)	118 (77.6)	0.329
CAT score, mean (SD)	25.4 ± 6.8	22.7 ± 6.7	0.107	25.2 ± 5.9	22.0 ± 7.2	0.044
^a^mMRC dyspnea scale, n (%)						
2–4	15 (78.9)	96 (79.3)	0.869	18 (81.8)	117 (77.0)	0.787
GOLD stage, A/B/C/D, n	1/7/3/8	2/54/23/42	0.332	0/9/4/9	5/83/24/40	0.095
GOLD C, D ratio, n (%)	11 (57.9)	65 (53.7)	0.379	13 (59.1)	64 (42.1)	0.134
Long-term oxygen therapy	7 (36.8)	26 (21.5)	0.117	6 (9.1)	27 (17.8)	0.380
Smoking amount, py, mean (SD)	43.1 ± 17.4	40.3 ± 15.8	0.506	45.0 ± 13.4	42.7 ± 17.8	0.604
BMI, kg/m^2^	21.9 ± 2.6	21.3 ± 2.9	0.481	21.5 ± 2.8	21.9 ± 3.8	0.666
Underlying comorbid conditions, n (%)						
Hypertension	7 (36.8)	71 (58.7)	0.140	15 (68.2)	103 (67.8)	0.969
Diabetes mellitus	12 (63.2)	59 (48.8)	0.190	8 (36.4)	71 (46.7)	0.362
Congestive heart failure	5 (26.3)	33 (27.3)	0.855	8 (36.4)	56 (36.8)	0.965
Chronic kidney disease	1 (5.3)	5 (4.1)	0.726	1 (4.5)	3 (2.0)	0.421
Hepatobiliary disease	0 (0.0)	3 (2.5)	0.306	1 (4.5)	3 (2.0)	0.421
Medications before an admission, n (%)						
Inhaled corticosteroids	11 (57.9)	72 (59.5)	0.951	13 (59.1)	86 (56.6)	0.824
Long-acting muscarinic antagonists	10 (52.6)	69 (57.0)	0.598	12 (54.5	76 (50.0)	0.690
Long-acting beta_2_ agonists	11 (57.9)	62 (51.2)	0.943	9 (40.9)	83 (54.6)	0.229
Functional parameters, %, mean (SD)						
Post-bronchodilator FEV_1_/FVC	49.0 ± 10.3	49.45 ± 13.6	0.962	48.3 ± 13.8	52.7 ± 13.2	0.093
Post-bronchodilator FEV_1_	58.7 ± 23.9	54.6 ± 21.0	0.606	55.5 ± 29.8	60.7 ± 22.5	0.217
Post-bronchodilator FVC	78.8 ± 23.2	72.3 ± 18.9	0.387	75.3 ± 24.7	76.5 ± 20.3	0.554
Mortality within 6 months, n (%)	15 (78.9)	19 (15.7)	< 0.001	9 (40.9)	14 (9.2)	< 0.001

*AECOPD* acute exacerbations of chronic obstructive pulmonary disease, *BMI* body mass index, *CAP* community-acquired pneumonia, *CAT* Chronic obstructive pulmonary disease assessment test, *FEV1* forced expiratory volume in 1 s, *FVC* forced vital capacity, *GOLD* Global Initiative for Chronic Obstructive Lung Disease, *mMRC* modified Medical Research Council, *n* number, *py* pack-years, *SD* standard deviation, *y* years old

^a^mMRC dyspnea scale consists in five statements that describe almost the entire range of dyspnea from none (grade 0) to almost complete incapacity (grade 4)

### The mortality rates for AECOPD patients who were readmitted

When we investigated the mortality rate of AECOPD patients readmitted between ≤30 days and > 30 days, the mortality rate during 6 months was the highest in AECOPD with CAP group who were readmitted ≤30 days (78.9% vs. 15.7% vs. 40.9% vs. 9.2%, *P* <  0.001) (Table 1). The causes of mortality were as follows; 30 patients with AECOPD (5 patients with CAP readmitted ≤30 days vs. 13 with CAP readmitted > 30 days vs. 3 patients without CAP readmitted ≤30 days vs. 9 AECOPD without CAP readmitted > 30 days), 18 pneumonias (10 vs. 3 vs. 2 vs. 3), 7 sepsis (0 vs. 3 vs. 1 vs. 3) and 2 pneumothoraxes (0 vs. 0 vs. 2 vs. 2).

### Laboratory and microbiologic findings

**Fig. 2:**
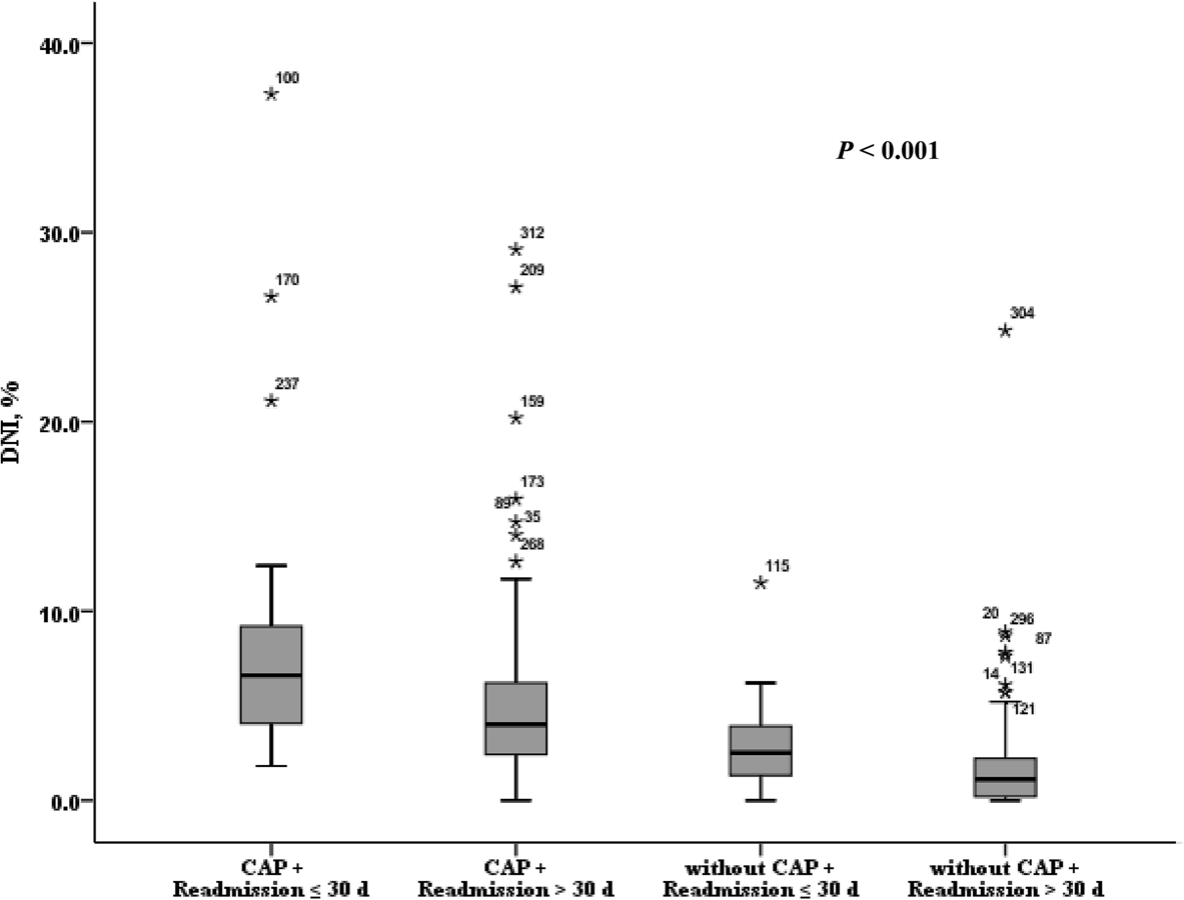
Fig. 2 shows mean DNI levels among four groups. Mean DNI values are 9.5 ± 9.2, 5.0 ± 4.6, 2.9 ± 2.6 and 1.7 ± 2.6%, respectively. It is significantly higher in AECOPD with CAP (readmitted ≤30 d) than without CAP. AECOPD = acute exacerbation of chronic obstructive pulmonary disease; CAP = community-acquired pneumonia; DNI = delta neutrophil index; d = days

At the time of admission, oxygen saturation, PaO_2_, PaCO_2_, hemoglobin, and procalcitonin were not significantly different, but WBC count (*P* <  0.001), serum DNI (*P* <  0.001) and hs-CRP levels (*P* <  0.001) showed significant differences among four groups (Table 2). Mean DNI values were 9.5 ± 9.2, 5.0 ± 4.6, 2.9 ± 2.6, and 1.7 ± 2.6% respectively, and were significantly higher in AECOPD with CAP (readmitted ≤30 days) than without CAP (Fig. 2).

**Table 2:** Laboratory and microbiologic findings between AECOPD with and without CAP

Variables (at the time of admission)	AECOPD with CAP		*P*-value	AECOPD without CAP		*P*-value
	Readmission ≤ 30 days (*n* = 19)	Readmission > 30 days (*n* = 121)		Readmission ≤ 30 days (*n* = 22)	Readmission > 30 days (*n* = 152)	
Laboratory findings, mean ± SD						
Oxygen saturation, %	88.3 ± 8.7	88.5 ± 10.0	0.926	86.8 ± 10.6	90.6 ± 8.9	0.153
PaO_2_, mm Hg	62.6 ± 15.1	62.5 ± 15.4	0.973	61.2 ± 16.5	64.4 ± 15.0	0.104
PaCO_2,_ mmHg	46.9 ± 30.1	38.2 ± 12.7	0.231	43.3 ± 18.5	40.1 ± 13.4	0.438
WBC, × 10^3^/μL	14.7 ± 6.9	13.0 ± 5.7	0.239	11.6 ± 6.4	10.2 ± 4.5	0.205
DNI, %	9.5 ± 9.2	5.0 ± 4.6	0.049	2.9 ± 2.6	1.7 ± 2.6	0.039
Hemoglobin, g/dL	12.0 ± 1.8	13.0 ± 1.8	0.029	12.6 ± 1.5	13.1 ± 1.9	0.198
hs-CRP, mg/dL	16.4 ± 8.3	15.9 ± 9.4	0.839	2.3 ± 2.3	3.2 ± 5.8	0.472
^a^Procalcitonin, mg/dL	8.1 ± 21.2	3.8 ± 7.4	0.467	1.3 ± 3.7	1.9 ± 6.1	0.753
Microbiologic findings, n (%)						
*Streptococcus pneumoniae*	7 (36.8)	47 (38.8)	0.868	1 (4.5)	2 (1.3)	0.335
*Staphylococcus aureus*	3 (15.8)	16 (13.2)	0.724	2 (9.1)	18 (11.8)	1.000
*Pseudomonas aeruginosa*	1 (5.3)	16 (13.2)	0.468	1 (4.5)	15 (9.9)	0.697
*Klebsiella pneumoniae*	2 (10.5)	5 (4.1)	0.297	1 (4.5)	10 (6.6)	1.000
^b^Others	0 (0.0)	5 (4.1)	1.000	0 (0.0)	2 (1.3)	1.000

*AECOPD* acute exacerbations of chronic obstructive pulmonary disease, *CAP* community-acquired pneumonia, *DNI* delta neutrophil index, *hs-CRP* high sensitive C-reactive protein, *n* number, *PaCO*_*2*_ arterial carbon dioxide partial pressure, *PaO*_*2*_ arterial oxygen partial pressure, *SD* standard deviation, *WBC* white blood cell

^a^Initial procalcitonin results were available for 81 patients in AECOPD with CAP and 94 patients in AECOPD without CAP

^b^Others were included *Mycoplasma pneumoniae* (2), *Legionella pneumophila* (1), *Haemophilus influenzae* (1) and *Acinetobacter baumannii* (1) in AECOPD with CAP, and *Legionella pneumophila* (1) and *Haemophilus influenzae* (1) in AECOPD without CAP, respectively

We identified causative organisms in 72.9% (102 out of 140) of AECOPD with CAP. *Streptococcus pneumoniae* was the most frequently isolated pathogen (38.6%); *Staphylococcus aureus* (13.6%), *Pseudomonas aeruginosa* (12.1%), *Klebsiella pneumoniae* (5.0%) and other pathogens (3.6%) were isolated, respectively (Table 2).

### Cumulative survival rates according to readmission duration and serum DNI level

**Fig. 3:**
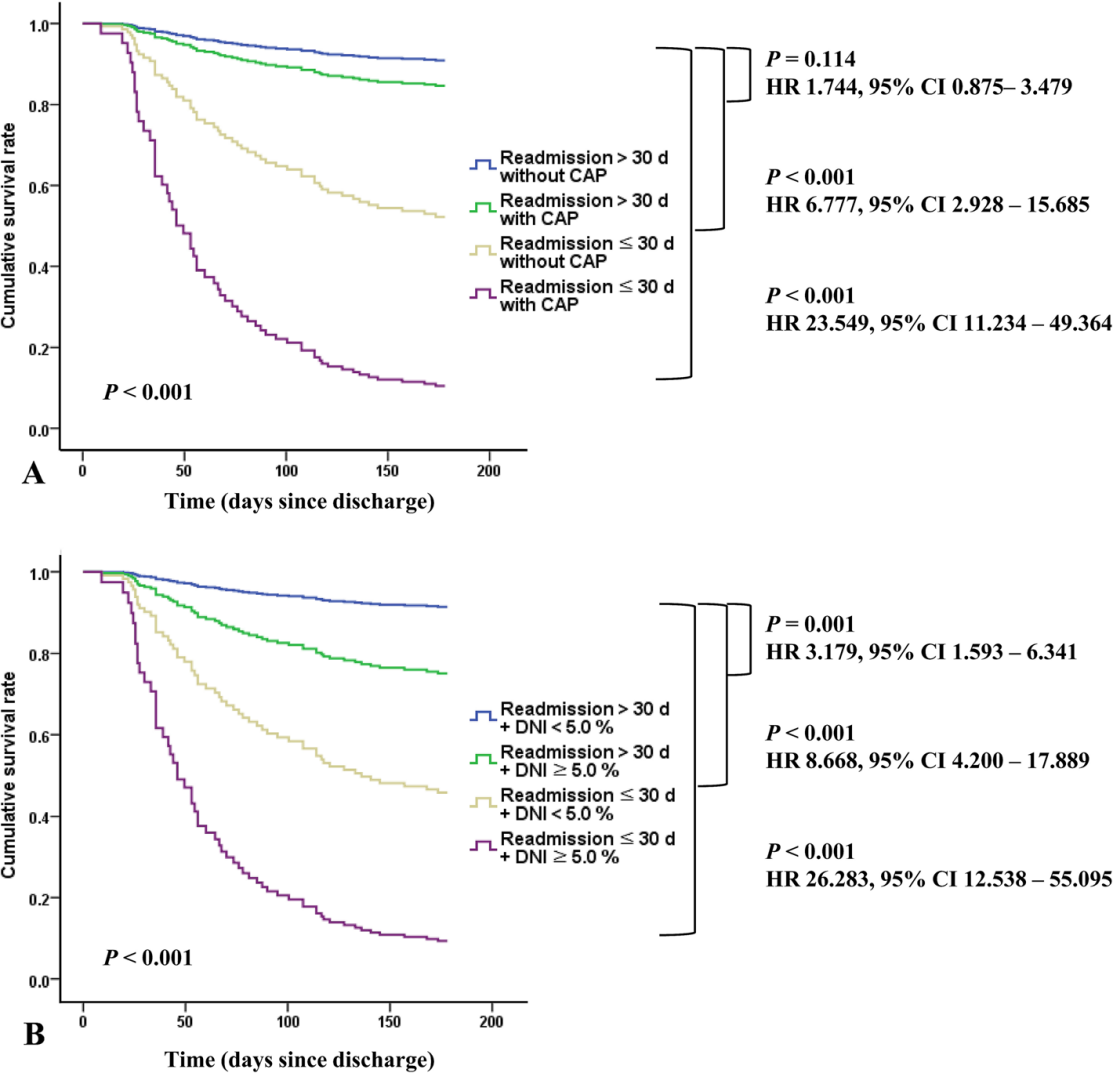
Fig. 3 shows cumulative survival rates during 6 months of AECOPD patients according to (**a**) readmission duration (< 30 vs > 30 d) and CAP, (**b**) readmission duration (≤ 30 vs > 30 d) and serum DNI level (< 3.5 vs ≥ 3.5%). **a** It shows the lowest cumulative survival rate in AECOPD with readmission ≤30 d and CAP (HR 23.549, 95% CI 11.234–49.364, *P* < 0.001). **b** It shows also the lowest cumulative survival rate in AECOPD with readmission ≤30 d and serum DNI ≥ 3.5% according to readmission duration and serum DNI level (HR 20.642, 95% CI 10.129–42.067, *P* < 0.001). AECOPD = acute exacerbation of chronic obstructive pulmonary disease; CAP = community-acquired pneumonia; DNI = delta neutrophil index; d = days

When we compared the cumulative survival rate of AECOPD patients according to readmission duration (≤ 30 vs > 30 days), AECOPD patients with readmission ≤30 days and CAP showed the lowest cumulative survival rate compared to other groups (*P* <  0.001) (Fig. 3a). When we compared the cumulative survival rate of AECOPD patients according to readmission duration (≤ 30 vs > 30 days) and DNI level (< 3.5 vs ≥ 3.5%), AECOPD patients with readmission ≤30 days and DNI ≥ 3.5% showed the lowest cumulative survival rate compared to other groups (*P* <  0.001) (Fig. 3b). Thus, the cumulative survival rate was lower as a serum DNI level (≥ 3.5%) was higher and readmission duration (≤ 30 days) was shorter.

### Prognostic factors associated with the mortality and ROC curve

To identify risk factors associated with the mortality of AECOPD patients who were readmitted, multivariate logistic regression analysis was performed using significant variables with *P* value < 0.05 by univariate analysis. Multivariate analysis using factors that were found to be significant by univariate analysis revealed that readmission duration ≤30 days (HR 7.879, 95% CI 4.554–13.632, *P* <  0.001); and serum DNI level (HR 1.086, 95% CI 1.043–1.131, *P* <  0.001) were significantly associated with the mortality of AECOPD patients during 6 months (Table 3). Pearson’s correlation coefficient (r value) of DNI (%) with hs-CRP was 0.433 (*P* < 0.001), DNI with PCT: 0.419 (*P* < 0.001), % DNI with PCO_2_: - 0.062 (*P* = 0.276), respectively.

**Table 3:** Prognostic factors associated with the mortality of AECOPD patients readmitted within 6 months

Variables	Univariate (mortality)			Multivariate (mortality)		
	95% CI	HR	*P*-value	95% CI	HR	*P*-value
Demographic						
GOLD group C-D	1.195, 3.549	2.060	0.009*	0.792, 2.863	1.506	0.212
Smoking amounts, py	0.995, 1.024	1.009	0.218			
Long-term oxygen therapy	0.261, 0.775	0.450	0.004*	0.303, 1.107	0.580	0.098
Readmission ≤30 d	6.679, 27.005	13.430	< 0.001*	4.554, 13.632	7.879	< 0.001*
Functional & laboratory						
Post-bronchodilator FEV1	0.977, 1.003	0.990	0.120			
WBC, /μL	1.000, 1.000	1.000	0.085			
DNI, %	1.064, 1.133	1.098	< 0.001*	1.043, 1.131	1.086	< 0.001*
hs-CRP, mg/dL	1.003, 1.048	1.025	0.029*	0.978, 1.032	1.005	0.736

*Indicates significance in univariate and multivariate analysis (*P* < 0.05)

*AECOPD* acute exacerbations of chronic obstructive pulmonary disease, *CI* confidence interval, *DNI* delta neutrophil index, *d* days, *GOLD* Global Initiative for Chronic Obstructive Lung Disease, *FEV*_*1*_ forced expiratory volume in 1 s, *hs-CRP* high sensitive C-reactive protein, *HR* hazard ratio, *py* pack-years, *WBC* white blood cell

**Fig. 4:**
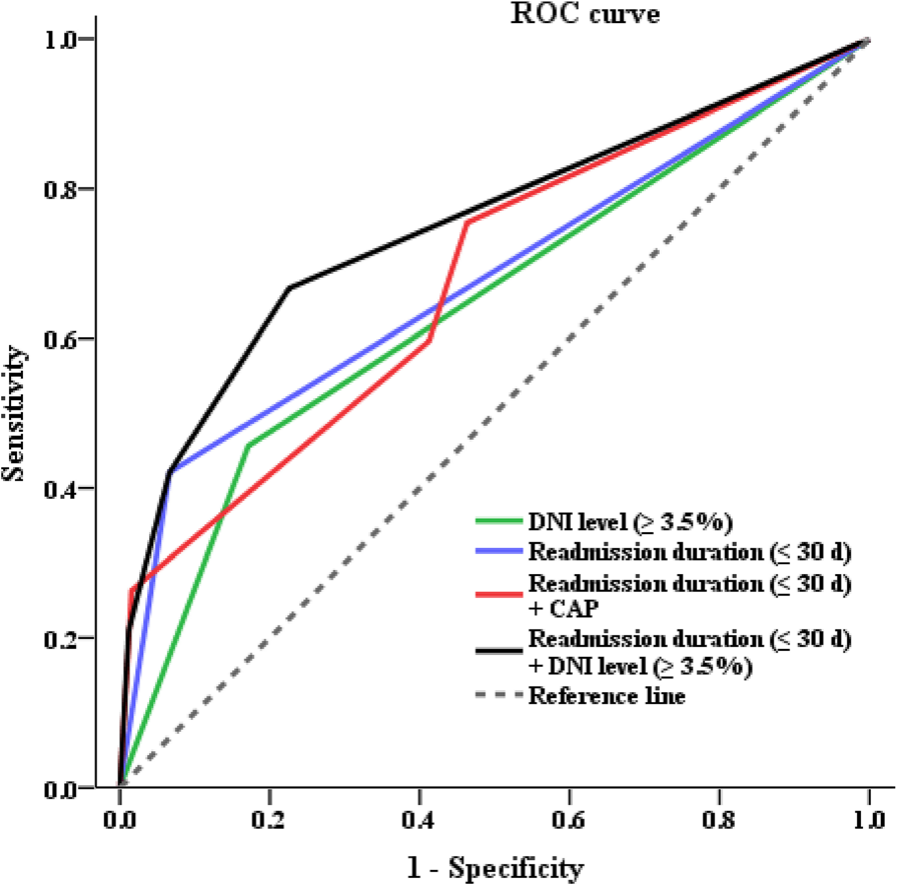
shows the ROC curve according to 1) readmission duration, 2) DNI, 3) readmission duration + DNI, and 4) readmission duration + CAP. AUC for readmission duration (≤ 30 d) + DNI level (≥ 3.5%) is 0.753 (95% CI 0.676–0.830, P < 0.001) with a sensitivity of 73.7% and a specificity of 67.3%; AUC for readmission duration + CAP 0.678 (95% CI 0.597–0.758), readmission duration 0.677 (95% CI 0.590–0.765), and DNI 0.654 (95% 0.573–0.735), respectively. AUC = area under the curve; CAP = community-acquired pneumonia; DNI = delta neutrophil index; d = days; ROC = receiver operating characteristic

The AUC for readmission duration (≤ 30 days) + DNI level (≥ 3.5%) was 0.753 (95% CI 0.676–0.830, *P* < 0.001) with a sensitivity of 73.7% and a specificity of 67.3%; AUC for readmission duration + CAP 0.678 (95% CI 0.597–0.758), readmission duration 0.677 (95% CI 0.590–0.765), and DNI 0.654 (95% 0.573–0.735), respectively (Fig. 4).

## Discussion

The most important point in this study was that AECOPD with CAP group who were readmitted ≤30 days and DNI ≥ 3.5% showed the lowest cumulative survival rate compared to the other groups. ROC curve demonstrated that DNI (≥ 3.5%) with readmission duration (≤ 30 days) can affect the mortality of AECOPD patients who were readmitted.

Currently, initial DNI with serum WBC and CRP which are commonly used markers is known to be useful for predicting inflammation and infection [23]. DNI, the difference between the leukocyte differentials measured in cytochemical myeloperoxidase channel and those assayed in the nuclear lobularity channel, reflects the fraction of circulating immature granulocytes [24, 25]. Previous study reported that granulocyte precursors less mature than bands can be a better predictor of infection than band neutrophil counts [26]. The diagnostic value of DNI (reflecting the fraction of circulating immature granulocytes) was superior to WBC, absolute neutrophil count or other widely available laboratory markers for severe sepsis/septic shock [25].

Many clinicians have studied the usefulness of blood biomarkers such as CRP and procalcitonin for early assessment of sepsis. Recently, systematic review and meta-analysis showed that the DNI has prognostic value in adults with sepsis and high DNI values tend to be associated with mortality in septic patients [15]. Several studies have been reported to use DNI for the diagnosis or prognosis of other infectious diseases including pneumonia, pulmonary tuberculosis, and acute prostatitis [23, 27, 28]. However, there are no reports on the usefulness of DNI in COPD patients. In our paper, we focused on the AECOPD patients who readmitted within 180 days of discharge.

The observational study showed that the readmission for AECOPD within 30 days is associated with a progressive increased long-term risk of death [5]. In this study, ROC curve showed that DNI was a useful biologic marker for predicting the mortality rate of AECOPD in addition to readmission within 30 days. DNI levels were higher in AECOPD with CAP patients than without CAP. This indicates that DNI levels are associated with infection even in AECOPD. However, the mortality rate was the highest in patients readmitted within 30 days, suggesting that readmission within 30 days had a greater impact on prognosis than DNI level.

Readmission within 30 days and serum DNI level were significantly associated with the mortality of readmitted AECOPD patients when we analyzed the risk factors using multivariate analysis. And when the cut off value of DNI level is set to 3.5%, readmission duration (≤ 30 days) and serum DNI level (≥ 3.5%) have shown to be more useful in predicting mortality than readmission duration (≤ 30 days) and CAP in ROC curve analysis.

Unfortunately, there are no studies showing the usefulness of DNI in AECOPD patients so far and it was a problem to determine the cutoff value of DNI to predict prognosis. Previous study [29] have shown that the DNI value of sepsis patients was 3.4% and our study confirmed that mean DNI value of AECOPD patients was 3.5%, so we set the cutoff value of DNI to 3.5%. Figure 3 also showed that AECOPD patients with readmission ≤30 days and DNI ≥ 3.5% showed significantly lower cumulative survival rate compared to other groups.

We considered why DNI levels are associated with mortality in AECOPD patients who were rehospitalized. Another study showed that DNI level at 72 h significantly correlated with mortality in patients with bacteremia [30], and increased DNI values at the time admission were significantly associated with severe sepsis/septic shock and overt disseminated intravascular coagulation (DIC) and the elevation of DNI value preceded the onset of organ/circulatory failure [25]. Our study showed that DNI levels were higher in AECOPD patients with CAP although we couldn’t confirm whether sepsis was associated with CAP. Patients with CAP are expected to have more patients with DIC, systemic inflammatory response syndrome or sepsis, which may have affected early readmission and mortality after discharge. However, there is no definitive study of the mechanism by which DNI affects mortality, and additional studies are needed.

We have some limitations in this study. First, the present study was performed in a single institution, selection bias may have influenced the significance of the present findings although the criteria of hospitalization were established, and thus a multicenter study is required to validate the results. Second, we did not accurately assess the patients with SIRS or sepsis in this study so could not clearly explain the reason for increased DNI in patients with CAP. In addition, there is no clear reason for why mortality is higher in the increased-DNI group in patients with early readmission and further research is needed. Third, DNI alone has weak predictive power against mortality in AUC curve and increases predictability when accompanied by early readmission in AECOPD patients. These results suggest that biologic markers such as DNI still have difficulties in predicting the mortality of AECOPD patients and should be considered with clinical factors such as early readmission.

However, this study is meaningful to confirm the higher mortality rate in the increased DNI group with early readmission among AECOPD patients who were readmitted.

## Conclusions

Our study showed that AECOPD with CAP group who were readmitted ≤30 days and DNI ≥ 3.5% showed higher mortality during 6 months compared to the other groups. DNI level can be used as a predictor of prognosis in AECOPD patients who were readmitted after discharge, further investigation will be necessary.

## Data Availability

All data generated or analysed during this study are included in this published article and its supplementary information files.

## Abbreviations

AECOPD: Acute exacerbations of chronic obstructive pulmonary disease
BMI: Body mass index
CAP: Community-acquired pneumonia
CAT: Chronic obstructive pulmonary disease assessment test
CI: Confidence interval
DNI: Delta neutrophil index
FEV_1_: Forced expiratory volume in 1 s
FVC: Forced vital capacity
GOLD: Global Initiative for Chronic Obstructive Lung Disease
HR: Hazard ratio
hs-CRP: high sensitive C-reactive protein
mMRC: modified Medical Research Council
PaCO_2_: Arterial carbon dioxide partial pressure
PaO_2_: Arterial oxygen partial pressure
ROC: Receiver operating characteristic
SD: Standard deviation
WBC: White blood cell

## References

[1] Vestbo J, Hurd SS, Agustí AG, Jones PW, Vogelmeier C, Anzueto A (2013). Global strategy for the diagnosis, management, and prevention of chronic obstructive pulmonary disease: GOLD executive summary. Am J Respir Crit Care Med.

[2] Wedzicha JA, Seemungal TAR (2007). COPD exacerbations: defining their cause and prevention. Lancet..

[3] Wedzicha JA, Brill SE, Allinson JP, Donaldson GC (2013). Mechanisms and impact of the frequent exacerbator phenotype in chronic obstructive pulmonary disease. BMC Med.

[4] Celli BR, MacNee W (2004). Standards for the diagnosis and treatment of patients with COPD: a summary of the ATS/ERS position paper. Eur Respir J.

[5] Guerrero M, Crisafulli E, Liapikou A, Huerta A, Gabarrús A, Chetta A (2016). Readmission for acute exacerbation within 30 days of discharge is associated with a subsequent progressive increase in mortality risk in COPD patients: a long-term observational study. PLoS One.

[6] Jacobs DM, Noyes K, Zhao J, Gibson W, Murphy TF, Sethi S (2018). Early hospital readmissions after an acute exacerbation of chronic obstructive pulmonary disease in the Nationwide readmissions database. Ann Am Thorac Soc.

[7] Donaldson GC, Seemungal TA, Bhowmik A, Wedzicha JA (2002). Relationship between exacerbation frequency and lung function decline in chronic obstructive pulmonary disease. Thorax.

[8] Schmidt SA, Johansen MB, Olsen M, Xu X, Parker JM, Molfino NA (2014). The impact of exacerbation frequency on mortality following acute exacerbations of COPD: a registry-based cohort study. BMJ Open.

[9] Yamauchi Y, Yasunaga H, Matsui H, Hasegawa W, Jo T, Takami K (2015). Comparison of clinical characteristics and outcomes between aspiration pneumonia and community-acquired pneumonia in patients with chronic obstructive pulmonary disease. BMC Pulm Med.

[10] Lu Z, Cheng Y, Tu X, Chen L, Chen H, Yang J (2016). Community-acquired pneumonia and survival of critically ill acute exacerbation of COPD patients in respiratory intensive care units. Int J Chron Obstruct Pulmon Dis.

[11] Andreassen SL, Liaaen ED, Stenfors N, Henriksen AH (2014). Impact of pneumonia on hospitalizations due to acute exacerbations of COPD. Clin Respir J.

[12] Festic E, Scanlon PD (2015). Incident pneumonia and mortality in patients with chronic obstructive pulmonary disease. A double effect of inhaled corticosteroids?. Am J Respir Crit Care Med.

[13] Baker CL, Zou KH, Su J (2013). Risk assessment of readmissions following an initial COPD-related hospitalization. Int J Chron Obstruct Pulmon Dis.

[14] Nantsupawat T, Limsuwat C, Nugent K (2012). Factors affecting chronic obstructive pulmonary disease early rehospitalization. Chron Respir Dis.

[15] Nahm CH, Choi JW, Lee J (2008). Delta neutrophil index in automated immature granulocyte counts for assessing disease severity of patients with sepsis. Ann Clin Lab Sci.

[16] Ahn C, Kim W, Lim TH, Cho Y, Choi KS, Jang BH (2018). The delta neutrophil index (DNI) as a prognostic marker for mortality in adults with sepsis: a systematic review and meta-analysis. Sci Rep.

[17] Hwang YI, Jung KS, Lim SY, Lee YS, Kwon NH (2013). A validation study for the Korean version of chronic obstructive pulmonary disease assessment test (CAT). Tuberc Respir Dis (Seoul).

[18] MacIntyre N, Huang YC (2008). Acute exacerbations and respiratory failure in chronic obstructive pulmonary disease. Proc Am Thorac Soc.

[19] Crisafulli E, Barbeta E, Ielpo A, Torres A (2018). Management of severe acute exacerbations of COPD: an updated narrative review. Multidiscip Respir Med.

[20] Lim WS, Baudouin SV, George RC, Hill AT, Jamieson C, Le Jeune I (2009). BTS guidelines for the management of community acquired pneumonia in adults: update 2009. Thorax.

[21] Müller B, Harbarth S, Stolz D, Bingisser R, Mueller C, Leuppi J (2007). Diagnostic and prognostic accuracy of clinical and laboratory parameters in community-acquired pneumonia. BMC Infect Dis.

[22] Mandell LA, Wunderink RG, Anzueto A, Bartlett JG, Campbell GD, Dean NC (2007). Infectious Diseases Society of America/American Thoracic Society consensus guidelines on the management of community-acquired pneumonia in adults. Clin Infect Dis.

[23] Cha YS, Lee KH, Lee JW, Kwon W, Lee SJ, Kang KS (2016). The usefulness of the Delta neutrophil index for predicting superimposed pneumonia in patients with acute decompensated heart failure in the emergency department. PLoS One.

[24] Kratz A, Maloum K, O’Malley C, Zini G, Rocco V, Zelmanovic D (2006). Enumeration of nucleated red blood cells with the ADVIA 2120 hematology system: an international multicenter clinical trial. Lab Hematol.

[25] Park BH, Kang YA, Park MS, Jung WJ, Lee SH, Lee SK (2011). Delta neutrophil index as an early marker of disease severity in critically ill patients with sepsis. BMC Infect Dis.

[26] Ardron MJ, Westengard JC, Dutcher TF (1994). Band neutrophil counts are unnecessary for the diagnosis of infection in patients with normal total leukocyte counts. Am J Clin Pathol.

[27] Jhun BW, Sim YS, Shin TR, Kim DG (2018). The utility of delta neutrophil index in differentiation of pulmonary tuberculosis from community acquired pneumonia. Sci Rep.

[28] Ahn HK, Koo KC, Chung BH, Lee KS (2018). Comparison of the delta neutrophil index with procalcitonin, erythrocyte sedimentation rate, and C-reactive protein as predictors of sepsis in patients with acute prostatitis. Prostate Int.

[29] Seok Y, Choi JR, Kim J, Kim YK, Lee J, Song J (2012). Delta neutrophil index: a promising diagnostic and prognostic marker for sepsis. Shock.

[30] Kim HW, Ku S, Jeong SJ, Jin SJ, Han SH, Choi JY (2012). Delta neutrophil index: could it predict mortality in patients with bacteraemia?. Scand J Infect Dis.

